# Disinfection and hand hygiene knowledge, attitude, and practices among childcare facilities staff during the COVID-19 pandemic in Anhui, China: a cross-sectional study

**DOI:** 10.3389/fpubh.2024.1335560

**Published:** 2024-04-04

**Authors:** Fang Chen, Qing Hua Xu

**Affiliations:** Anhui Center for Disease Control and Prevention, Hefei, Anhui, China

**Keywords:** disinfection, hand hygiene, childcare facilities, knowledge, attitude, practice, COVID-19

## Abstract

**Objective:**

This study aimed to investigate the knowledge, attitude, and practice (KAP) regarding disinfection and hand hygiene, along with associated influencing factors among childcare facilities staff during the COVID-19 pandemic in Anhui, and to provide information for developing disinfection and hand hygiene strategies for childcare facilities.

**Methods:**

A web-based cross-sectional study was conducted among Anhui Province residents in China in September 2020. In this study, 60 childcare facilities in two cities of Anhui Province were selected using the convenient sampling method for questionnaires. The questionnaires were distributed through a web-based platform. The disinfection and hand hygiene KAP scores among childcare facilities staff were calculated, and their influencing factors were analyzed. The accuracy rates of knowledge, attitude, and practice of behavior were calculated and analyzed.

**Results:**

A total of 1,029 participants were included in the study. The disinfection and hand hygiene knowledge, attitude and practice ranged from approximately 5 to 23, 1 to 5, 3 to 13, respectively. The score of urban areas was higher than that of rural areas. Higher education levels and more years of working were associated with higher scores. Additionally, staff who received training or supervision had higher scores than those without. The categories with the lowest knowledge accuracy rate (46.3%), lowest attitude accuracy rate (4.2%), and “always” practice rate (5.3%) among childcare facility staff were all related to the question categories concerning the appropriate range of disinfectants for use. The accuracy rates of hand hygiene knowledge and attitude among the childcare facility staff were high (83.7%-99.6%), but the “always” practice rate was in the middle range (63.0%).

**Conclusion:**

The disinfection and hand hygiene knowledge among childcare facilities staff was inadequate during the COVID-19 pandemic in Anhui. Continuous implementation of education and training, particularly in rural areas, is essential. Establishing a monitoring system to assess usage effectiveness and adverse reactions in China is critical. Interventions should focus on increasing compliance with hand hygiene practices. Further research should explore the training and intervention of disinfection and hand hygiene, the safety of disinfection measures, and more operational hand hygiene methods in childcare facilities.

## Introduction

Childcare facilities, catering to children aged 0 to 6 years old, are often hotspots for clusters or outbreaks of acute gastroenteritis, hand, foot, and mouth disease, and influenza (1–3). During the COVID-19 pandemic, clusters of COVID-19 infections have occurred in childcare facilities (4). Disinfection is an important measure to cut off transmission, and hand hygiene is the most important, effective, and economical measure to prevent and control infection rates. The World Health Organization (WHO) recommends environmental cleaning, disinfection, and hand hygiene for the prevention and control of infectious diseases (5). However, studies have revealed that the implementation of these measures has not been satisfactory. Additionally, the unqualified environment disinfection and hand hygiene among children and teachers have been identified as risk factors for enterovirus infection in childcare facilities (6).

During the COVID-19 pandemic, the prevention and control measures of infectious diseases, such as disinfection and hand hygiene, have been promoted by many countries. Although residents exhibited good knowledge and attitude toward these measures, the behavior status was not satisfied. Some studies indicated that factors such as gender, age, and education level influenced behavior, with good knowledge and attitude serving as prerequisites for proper behavior (7, 8). Caregivers and teachers serve as a critical barrier in safeguarding children's health, while simultaneously emphasizing the importance of their own self-protection, and also play an important role for children getting and implementing health measures (9, 10). However, limited studies have been conducted on knowledge, attitude, and practice (KAP) toward disinfection and hand hygiene among childcare facilities staff. Some studies on knowledge and its intervention have exhibited differences in the knowledge rate among childcare facilities staff in different areas, with significant increases observed in knowledge rates to a high level after training (11, 12). Hand hygiene behaviors among childcare facilities staff were related to their knowledge, perception, habits, and interactions with children (13). During the COVID-19 pandemic, a KAP study on the prevention and control of hand, foot, and mouth disease among childcare facilities staff, including several disinfection and hand hygiene measures, exhibited that the staff possessed a low level of knowledge, but their attitude was positive. While a few staff practiced at a high level (14), it is suggested that the characteristics of disinfection and hand hygiene KAP among childcare facilities staff differed from those of the general public probably.

In China, childcare facilities have been listed as important institutions for the prevention and control of COVID-19 by the government. These institutions are required to perform environmental cleaning, disinfection, and hand hygiene (15). However, there is a lack of systematic surveillance and studies on KAP of childcare facilities staff. Understanding the disinfection and hand hygiene KAP among childcare facilities staff and discussing the improving measures are important steps to enhance environmental disinfection and hand hygiene as well as prevention and control of infectious disease among childcare facilities staff. This study was conducted based on a survey project of the disinfection practices in childcare facilities in Anhui Province in September 2022. The study aimed to investigate the disinfection and hand hygiene KAP among childcare facilities staff in two cities and analyze the influencing factors. This study also identified the gaps mainly among the knowledge, attitude, and practice, but not the scores of every part of knowledge, attitude, and practice, or total score and the relationship of the three as usual, to provide a scientific basis for the implementation and evaluation of intervention activities at every link in the future.

## Materials and methods

### Study setting and design

This study was a web-based cross-sectional study conducted among residents of Anhui Province, China, during the COVID-19 pandemic, in September 2020.

### Sampling and sample size

A stratified sampling method was employed to select one city from the south and another from the north of Anhui Province. In each selected city, 3 districts were chosen and, within each district, 10 childcare facilities in each district were selected using a convenient sampling method to conduct the study. The childcare facilities included in this study comprised nurseries and kindergartens of all levels and types, catering to children aged 0–6 years.

Assuming that 80%(p) of staff were at a high level of disinfection and hand hygiene knowledge (16, 17), with a type Ierror (α) of 0.05 and the permissible error (d) of 0.1 p, considering 10% expected missing and incomplete responses in the online survey and the childcare facilities administrators participating in distribution work. The minimum sample size was 120 per district, with the sample size calculation formula (*n* = ${\text{t}}_{0.05}{\;}^{2}\text{P}(1-\text{P})/{\text{d}}^{2}$) for the status study.

### Questionnaire preparation and description

The questionnaires focused on disinfection and hand hygiene were developed based on existing research literature. The content validities of the questionnaire were reviewed by four experts (three experts in disinfection and one epidemiologist; Senior Doctor), each of whom provided separate comments. The researcher collected these comments and revised the questionnaire accordingly. A pre-survey was conducted among 20 staff members of childcare facilities staff, who completed the questionnaire and reported whether it was easy to understand. Based on their feedback, modifications were made and the formal questionnaire was formed.

The questionnaire primarily included the following contents: socio-demographic data and the method of access to knowledge (11 questions), disinfection and hand hygiene knowledge (8 questions), attitude (5 questions), and behavior (7 questions) on the purpose, object, method, timing of disinfection and hand hygiene. There were 31 questions in total.

The questionnaire scoring system is described as follows: first, in the knowledge questions, each correct response was scored “1,” while incorrect response/not sure was scored “0.” Second, in the attitude questions, a positive (correct) response was scored “1,” while a negative/neutral response was scored “0.” Third, in the practice questions, for multiple-choice questions, the number of correct responses was classified as positive, moderate, and negative behavior, scored “2”, “1,” and “0”, while for single-choice questions, the behavior implemented “always”, “sometimes,” and “never” were scored “2”, “1,” and “0”, respectively. The total score for KAP was 42, incorporating 23 points for knowledge, 5 points for attitude, and 14 points for practice.

Cronbach's alpha test was conducted using SPSS version 20.0 to check the reliability of the questionnaire and was found to be 0.765 (The multiple choice questions were split into several corresponding single choice data when exported from the WJX platform automatically).

### Data collection

The questionnaires were distributed to respondents via the questionnaire platform powered by www.wjx.cn (WJX) and WeChat app in September 2022, after being approved by the local education administration. Informed consent was obtained from all participants. Within each childcare facility, three staff members from the departments of administrative management, childcare, healthcare, cleaning and disinfection, and food and beverage support completed the questionnaires; all staff answered if there were fewer than three staff members in a department. A total of 1,036 respondents were obtained, with 1,029 valid responses, resulting in a response rate of 99.3%.

The childcare facilities' character data (urbanicity, owned) were provided by local education administration and were assigned to response data exported from the WJX platform by researchers, depending on the name of the childcare facilities in response data, to avoid misunderstanding of the facility information by staff.

### Data analysis

All the collected data in the “WJX” platform were exported to a master Excel spreadsheet (Microsoft Office 2007) for cleaning and coding before being imported into Statistical Package for Social Sciences (IBM SPSS20.0). Numbers and percentages were used to describe the categorical variables. When calculating the accuracy rate, the multiple-choice question was considered correct if it received a total score. The median (Q1, Q3) was used to describe continuous variables such as the KAP score of different groups. The Mann–Whitney test and the Kruskal–Wallis test were used for analyzing knowledge scores among different groups. The chi-square tests were used for analyzing trained or supervised percentages among various groups. A *P*-value of < 0.05 was considered statistically significant.

### Ethical considerations

This study was approved by the Ethics Committee of Anhui Provincial Center for Disease Control and Prevention. The title and purpose of the study were presented at the beginning of the questionnaire. The contents did not include any personal identifiers. Informed consent was obtained from all participants in this study. Privacy and confidentiality of all information were strictly maintained.

## Results

### Information on survey objects

A total of 1,029 participants in 60 childcare facilities were included in the study. Of these, 97.7% of participants were female participants, mainly aged between 17 and 66 years, 46.4% were below 30 years old, 41.0% held junior college diplomas, 87.1% were majors in childcare and education, and 42.3% were working for 3 to 9 years. Additionally, 56.1% and 43.9% of participants resided in Bozhou City and Huangshan City, respectively; 70.8% were in urban areas; and 73.8% were in public childcare facilities (Table 1).

**Table 1:** The scores of disinfection and hand hygiene KAP among childcare facilities staff, Anhui Province, China, September 2022 (*n* = 1,029).

**Characteristic**		**Staff N (%)**	**Knowledge score median (Q1, Q3)**	**P**	**Attitude score median (Q1, Q3)**	**P**	**Practice score median (Q1, Q3)**	***P***
**Region**								
	Bozhou	577 (56.1)	22.00 (21.00,22.00)	< 0.001	4.00 (4.00,4.00)	0.001	11.00 (10.00,12.00)	0.141
	Huangshan	452 (43.9)	22.00 (21.00,23.00)		4.00 (4.00,4.00)		11.00 (10.00,12.00)	
**Urbanicity**								
	Urban	729 (70.8)	22.00 (21.00,23.00)	0.003	4.00 (4.00,4.00)	0.741	11.00 (10.00,12.00)	0.248
	Suburb	300 (29.2)	22.00 (21.00,22.00)		4.00 (4.00,4.00)		11.00 (10.00,12.00)	
**Owned**								
	Public	759 (73.8)	22.00 (21.00,23.00)	0.686	4.00 (4.00,4.00)	0.042	11.00 (10.00,12.00)	0.164
	Privately	270 (26.2)	22.00 (21.00,23.00)		4.00 (4.00,4.00)		11.00 (10.00,12.00)	
**Gender**								
	Male	24 (2.3)	22.00 (18.75,22.75)	0.634	4.00 (4.00,4.00)	0.759	12.00 (10.25,12.00)	0.408
	Female	1005 (97.7)	22.00 (21.00,23.00)		4.00 (4.00,4.00)		11.00 (10.00,12.00)	
**Age (years)**								
	17-29	477 (46.4)	22.00 (21.00,22.00)	0.078	4.00 (4.00,4.00)	0.706	11.00 (10.00,12.00)	< 0.001
	30-39	299 (29.1)	22.00 (21.00,23.00)		4.00 (4.00,4.00)		11.00 (10.00,12.00)	
	40-49	152 (14.8)	22.00 (21.00,22.00)		4.00 (4.00,4.00)		12.00 (11.00,12.00)	
	50+	101 (9.8)	22.00 (20.00,23.00)		4.00 (4.00,4.00)		12.00 (10.00,12.00)	
**Education**								
	University graduate	411 (39.9)	22.00 (21.00,23.00)	0.026	4.00 (4.00,4.00)	0.048	11.00 (10.00,12.00)	0.034
	Junior college	422 (41.0)	22.00 (21.00,23.00)		4.00 (4.00,4.00)		11.00 (10.00,12.00)	
	High school graduate	73 (7.1)	22.00 (21.00,23.00)		4.00 (4.00,4.00)		11.00 (9.50,12.00)	
	High school or less	123 (12.0)	22.00 (20.00,22.00)		4.00 (4.00,4.00)		12.00 (11.00,12.00)	
**Profession**								
	Childcare and educate	896 (87.1)	22.00 (20.00,22.00)	0.344	4.00 (4.00,4.00)	0.317	12.00 (11.00,12.00)	0.002
	Healthcare	65 (6.3)	22.00 (21.00,23.00)		4.00 (4.00,4.00)		11.00 (10.00,12.00)	
	Others	68 (6.6)	22.00 (20.00,22.00)		4.00 (4.00,4.00)		11.00 (10.00,12.00)	
**Years of working**								
	0-2	335 (32.6)	22.00 (20.00,23.00)	0.039	4.00 (4.00,4.00)	0.070	11.00 (10.00,12.00)	0.145
	3-9	435 (42.3)	22.00 (21.00,23.00)		4.00 (4.00,4.00)		11.00 (10.00,12.00)	
	10+	259 (25.2)	22.00 (21.00,23.00)		4.00 (4.00,4.00)		11.00 (10.00,12.00)	
**Trained and supervised**								
	Yes	984 (95.6)	22.00 (21.00,23.00)	0.001	4.00 (4.00,4.00)	0.042	11.00 (10.00,12.00)	0.040
	No	45 (4.4)	21.00 (19.00,22.00)		4.00 (4.00,4.00)		11.00 (8.00,12.00)	

### Disinfection and hand hygiene KAP score and influencing factors

The disinfection and hand hygiene knowledge score ranged between approximately 5 and 23. The different characteristics of staff were compared to determine the influencing factors in the scores of disinfection and hand hygiene knowledge. Scores in Huangshan were significantly higher than that in Bozhou; in urban areas, scores were significantly higher than that in suburban areas (*P* < 0.001 and *P* = 0.003, respectively). Higher education levels and the more years of working were significantly associated with higher scores (*P* = 0.026 and *P* = 0.039, respectively). The scores for staff with training or supervision were significantly higher than those for those without (*P* = 0.001). Scores of staff, whether in public or privately owned facilities and irrespective of gender or professions, were found to be non-significant.

The disinfection and hand hygiene attitude scores ranged from approximately 1 to 5. Scores of staff were different significantly with different regions, whether facilities were public or privately owned, and whether they received education and training or supervision (*P* = 0.001, *P* = 0.042, *P* = 0.048, and *P* = 0.042, respectively). Scores of staff with urban or suburb, gender, ages, professions, and years of working were found to be non-significant.

The disinfection and hand hygiene practice score ranged from approximately 3 to 13. Scores of staff with different ages, educations, professions, and training or supervision were significantly different (*P* < 0.001, *P* = 0.034, *P* = 0.002 and *P* = 0.040, respectively). Scores of staff with different regions, urban or suburban settings, public or privately owned facilities, gender, or years of working were found to be non-significant (Table 1).

### Disinfection and hand hygiene knowledge questionnaire

Among the questions of knowledge, the highest accuracy was in the category of “Know the purpose of disinfection was cutting off transmission” (99.9%), followed by “Know that disinfectant should be used within the expiration date” (99.6%), and “Know that surfaces of environmental objects need to be cleaned at first and then disinfected” (98.5%). The lowest accuracy category was “Know that alcohol disinfectants could not be used for all infectious diseases” (46.3%) (Table 2).

**Table 2:** The categories of disinfection and hand hygiene knowledge and attitude among childcare facilities staff, Anhui Province, China, September 2022 (*n* = 1,029).

**Question**		**Correct N (%)**
**Knowledge**		
	1. Know the purpose of disinfection was cutting off transmission	1,028 (99.9)
	2. Know the objects need to be disinfected (select all five)^a^	930 (90.4)
	3. Know the method to disinfect surfaces (select all four)^b^	713 (69.3)
	4. Know that surfaces of environmental objects need to be cleaned at first and then disinfected	1,014 (98.5)
	5. Know that disinfectant should be used within the expiration date	1,025 (99.6)
	6. Know that it is better to wash your hands with running water and hand sanitizer	861 (83.7)
	7. Know the timing of hand hygiene (select all nine)^c^	916 (89.0)
	8. Know that alcohol disinfectants could not be used for all infectious diseases	476 (46.3)
**Attitude**		
	1. Consider that disinfection is important for the prevention and control of infectious diseases	1,025 (99.6)
	2. Consider that there is no need for large-scale outdoor disinfection during the pandemic	43 (4.2)
	3. Consider that washing hands can reduce the chance of infection	1,004 (97.6)
	4. Be willing to instruct family members to wash their hands with running water and hand sanitizer	1,025 (99.6)
	5. Consider that the 6-step wash makes your hands cleaner	997 (96.9)

^a^The five options of disinfection objects are door handles, switch buttons, tables and chairs, faucets, and toilets. ^b^The four options of disinfection methods are wiping with chlorine disinfectant, spraying with chlorine disinfectant, wiping with 75% ethanol, and ultraviolet irradiation. ^c^The nine options of timing of hand hygiene are before eating, before caring for the older adult and infants, before making food, after going to the toilet, after coughing with hands over mouth and nose, after caring for a sick person, after handling garbage and dirt, and after touching surfaces with high frequency of contact during an epidemic of infectious diseases.

### Disinfection and hand hygiene attitude questionnaire

Among the questions of attitude, the highest accuracy of childcare facilities staff was “Consider that disinfection is important for the prevention and control of infectious diseases” and “Be willing to instruct family members to wash their hands with running water and hand sanitizer” (97.6% and 99.6% respectively). The lowest accuracy was “Consider that there is no need for large-scale outdoor disinfection during the pandemic” (46.3%) (Table 2).

### The technical support of disinfection and hand hygiene knowledge

Comparing the staff trained or supervised or not in different characteristics, the percentage of staff receiving training or supervision in urban areas was significantly higher than that in rural (χ^2^ = 24.913, P < 0.001) (Table 3).

**Table 3:** The technical support of disinfection and hand hygiene knowledge among childcare facilities staff, Anhui Province, China, September 2022 (*n* = 1,029).

**Characteristic**		**Trained or supervised N (%)**	**χ^2^**	***P***
**Region**			
	Bozhou	547 (94.8)	2.144	0.143
	Huangshan	437 (96.7)		
**Urbanicity**			
	Urban	712 (97.7)	24.913	< 0.001
	Suburb	272 (90.7)		
**Ownership rights**			
	Public	726 (95.7)	0.004	0.947
	Private	258 (95.6)		

### The method of access to disinfection and hand hygiene knowledge

The primary source of disinfection and hand hygiene knowledge for childcare facilities staff was training organized by facilities themselves (84.8%). Following that were training organized by superior institutions (77.9%). Other sources included WeChat accounts (77.7%), technical departments (70.8%), Television (59.9%), websites (58.5%), poster brochures (57.4%), and radio (50.0%).

### Disinfection and hand hygiene practice questionnaire

Among the childcare facilities staff, 80.9% reported having prepared more than two kinds of disinfectants at home since 2020, 92.7% disinfected more than three kinds of objects for prevention, 74.0% cleaned first and then disinfected always, 74.4% wore masks and gloves when using chlorine disinfectant always, 89.7% washed hands when coming back from public places always, 63.0% washed hands with 6 steps always, while 5.3% never “sprayed alcohol to disinfect air since 2020” (Table 4).

**Table 4:** The questions of disinfection and hand hygiene practices among childcare facilities staff, Anhui Province, China, September 2022 (*n* = 1,029).

**Question**		**Staff N (%)**
**1. Prepared disinfectants at home since 2020**		
	More than 2 kinds^a^	832 (80.9)
	1-2 kinds	193 (18.8)
	None of the above	4 (0.3)
**2. Disinfected objects for prevention since 2020**		
	More than 2 kinds^b^	954 (92.7)
	1-2 kinds	69 (6.7)
	None of the above	6 (0.6)
**3. Cleaned, then disinfected since 2020**		
	Always	761 (74.0)
	Sometimes	267 (25.9)
	Never	1 (0.1)
**4. Wore masks and gloves when using chlorine disinfectant since 2020**		
	Always	766 (74.4)
	Sometimes	254 (24.7)
	Never	9 (0.9)
**5. Sprayed alcohol to disinfect air since 2020**		
	Never	55 (5.3)
	Sometimes	974 (94.7)
**6. Washed hands when coming back from public places since 2020**		
	Always	923 (89.7)
	Sometimes	106 (10.3)
	Never	0 (0)
**7. Washed hands with 6 steps since 2020**		
	Always	648 (63.0)
	Sometimes	372 (36.2)
	Never	9 (0.8)

^a^The five kinds of disinfection products are chlorine disinfectant, 75% alcohol, hand sanitizer, disinfectant wipes, and ultraviolet lamps. ^b^The five kinds of disinfection objects are door handles, switch buttons, tables and chairs, faucets, and toilets.

## Discussion

The results of this study showed a significant correlation between the higher educational level of the childcare facilities staff and their higher score, which was consistent with the survey results of resident adults and children's parents (8, 18). This result suggests that staff with a higher degree of education may possess a better understanding and mastery of knowledge. The results of this study showed that the education level of the childcare facilities staff mainly consisted of junior college (41.0%) and university graduates (39.9%), which was consistent with the survey results in China (12, 19). However, this finding was inconsistent with the research in other countries, such as Singapore, where the education of the staff mainly consisted of secondary school and diploma courses (14, 20). Although the effect of the educational level of childcare facilities staff on the implementation of hygiene measures among children has not been reported, some studies showed that the education level of parents was positively correlated with the hand hygiene behavior of students (21). Childcare facilities staff play a vital role in taking care and teaching children, akin to children's parents. The educational level of staff should be considered in the health care work of the childcare facilities. This study showed that the childcare facilities staff were predominantly female individuals (97.7%) and mainly aged from 17 to 29 years old (46.4%), which was consistent with other studies (12, 14, 22). This demographic trend may be attributed the characteristics and caring needs of children (20). However, this study showed that there was no significant difference in the knowledge scores of the staff by gender and age. This aspect was inconsistent with the research of the resident, which showed that the older and female individuals were more compliant with COVID-19 prevention and control measures (7). The results of this study also showed that the more the years of working, the higher the score significantly. The scores of staff with training or supervision were significantly higher than those without training or supervision. This difference may be due to different health promotion models between the public and childcare facilities staff. This study revealed that the training rate was 84.8% in facilities and 77.9% by higher level organizations among the staff. Organized training was more targeted and definitely showed effectiveness in improving the disinfection and hand hygiene knowledge of childcare facilities staff.

The results of this study showed that the disinfection and hand hygiene knowledge scores of staff were significantly different between the two cities, and the scores in urban childcare facilities were significantly higher than those in rural childcare facilities. This finding was partly consistent with the survey results of public adults in America and Saudi Arabia, which demonstrated that it was different among different cities because of the COVID-19 situation probably. At the same time, there was no difference between rural and urban areas (7, 16). The difference between rural and urban areas in this study was consistent with the survey results of hand hygiene knowledge of kindergarten teachers in Zhejiang, Shanghai, China, during the COVID-19 pandemic (19, 22). This result may be due to the low educational level of staff from rural childcare facilities in China, and they were short of the ability and opportunity to acquire relevant knowledge (19). This study also showed that the percentage of staff in rural childcare facilities who received disinfection and hand hygiene technical training or supervision was significantly lower than that in urban areas. However, the spread rate of infectious disease clusters in rural schools was higher than that in urban areas, while the qualified rate of disinfection in rural schools was lower than that in urban areas (23, 24). Therefore, childcare facilities in rural areas were critical segments to improve the control and prevention strategies for infectious disease in China. Moreover, the results of this study showed that scores of staff with different regions, public or privately owned, education, and training or supervision were significantly different. Scores of staff with different ages, educations, professions, and training or supervision were significantly different. The influencing factors that influenced attitude and practice were inconsistent with that of knowledge, except for training and supervision. This aspect highlighted the importance of training and supervision effort and should be more targeted.

The results of this study demonstrated that the lowest knowledge accuracy rate of childcare facilities staff was about the usable range of disinfectants: The accuracy rate of “Know that alcohol disinfectants could not be used for all infectious diseases” was 46.3%. The lowest attitude and behavior accuracy were consistent with that of knowledge. “Consider that there is no need for large-scale outdoor disinfection during the pandemic” was the lowest accuracy rate for attitude (4.2%), and “Never sprayed alcohol to disinfect air since 2020” was the lowest accuracy rate for practice (5.3%). Although the Chinese government promoted work requirements such as the “seven No's” principle of disinfection and the application scope and use method of common disinfectants (25), this study showed inadequate implementation in childcare facilities. According to a survey among 154 countries, China is one of the countries with the highest use of disinfectants during the COVID-19 pandemic, alcohol-based and chlorine-based disinfectants were reported to be the most widely used. The most commonly reported health problems, including skin and respiratory issues, were related to the two disinfectants mentioned earlier (26). The reasons for health problems included misuse and overuse of disinfectants (26, 27). During the COVID-19 pandemic, alcohol poisoning accounted for 8.5% of children in the pediatric emergency department of a hospital in Turkey, indicating a higher incidence compared to before the pandemic (28). This study highlighted the dangers posed to both staff and children if disinfectants were misused. In the United Kingdom and the United States, there are monitoring systems for poisoning caused by exposure to disinfectants (27, 29), it is helpful to evaluate and provide guidance to the public on the proper use of disinfectants. It is suggested that China also needs to establish and improve a monitoring system covering the use of disinfectants, disinfection effectiveness, and adverse reactions.

The results of this study showed that although the accuracy rate of hand hygiene knowledge among the childcare facility staff was high—“Know that it is better to wash your hands with running water and hand sanitizer” and “Know the timing of hand hygiene” were 83.7% and 89.0%, respectively—the attitude of staff was positive too. The support rate of “Consider that the 6-step wash makes your hands cleaner” was 96.9%, the “always” practice rate of “Washed hands when coming back from public places from 2020” was 89.7%, which was consistent with the survey results of rural adults from 3 provinces in China (17), and it was higher than the rate of adult residents from Ghana (61.5%−66.5%) (8). However, the practice rate of “ Wash hands with six steps since 2020” was not high, with “always” measured at 63.0%. The rate was lower than the implementation rate (79%−87%) of adults who “always” washed their palms, back of hands, between the fingers, fingertips, and thumbs during the partial block in Saudi Arabia 2020 (30). It may be related to the trend of the pandemic, and the difference between respondents. Furthermore, washing hands with six steps needs more time, as the rate of washing at least 20 times was not high (51.7%) in rural adults from 3 provinces in China (17). This finding is similar to the responsibilities of medical workers as childcare facility staff take care of children, and their hand hygiene compliance should be improved. Some studies investigated disinfection and hand hygiene knowledge in childcare facilities. While the intervention methods varied significantly, such as training and publicity, facility improvement, and interest stimulation, the effectiveness remained inconsistent (31–33). To improve the operability of hand hygiene, some studies in medical institutions showed that three-step techniques with hand rubbing (1. covering all surfaces of the hands, 2. rotationally rubbing the fingertips in the palm of the alternate hand, and 3. rotationally rubbing both thumbs) resulted in higher compliance with both hand hygiene indications and technique compared to the six steps. As the results of the microbiological analyses exclude inferiority, the conventional six steps could be safely replaced by a simpler hand hygiene technique. Three-step techniques were simpler, faster, and easier to popularize among the public (34). However, whether three steps with water and handwashing work similarly or not, and the effects on infectious disease control and prevention in the community need to be studied. Moreover, childcare facilities should be accompanied by specifically structured, model surveillance studies that further clarify outstanding questions about infectious disease events and hygiene control (10).

## Limitation

In this study, a stratified multi-stage and convenient sampling method was adopted to select districts and childcare institutions, but not randomly. The reason for this was the lack of a perfect disinfection work monitoring system in childcare facilities in our province, leading to varying study conditions and degree of cooperation among different districts. As a result, the knowledge accuracy rate, attitude support rate, and compliance level of childcare facilities staff in this study may be better than the provincial average level. Therefore, the analysis and discussion were mainly conducted from the perspective of the facilities' character, working modes, and other notable limitations.

## Conclusion

During the COVID-19 pandemic, the disinfection and hand hygiene knowledge among childcare facilities staff was found to be inadequate, especially in rural areas where training and publicity should be strengthened. However, the attitude and knowledge of the staff were mostly consistent. Hand hygiene compliance should be improved. We recommend establishing a comprehensive monitoring system that includes the usage, effectiveness, and adverse reactions related to disinfection, along with evaluating and guiding the public in the proper use of disinfectants in China. Furthermore, effective intervention measures should be taken to enhance staff compliance with hand hygiene. Further research should be considered on the training and intervention of disinfection and hand hygiene, the safety of disinfection measures, and more operational hand hygiene methods in childcare facilities.

## Data availability statement

The original contributions presented in the study are included in the article/supplementary material, further inquiries can be directed to the corresponding author: hscf119@163.com.

## Ethics statement

Ethical approval was not required for the study involving humans in accordance with the local legislation and institutional requirements. The studies were conducted in accordance with the local legislation and institutional requirements. Written informed consent to participate in this study was not required from the participants in accordance with the national legislation and the institutional requirements.

## Author contributions

FC: Conceptualization, Data curation, Formal analysis, Funding acquisition, Investigation, Methodology, Project administration, Resources, Software, Supervision, Validation, Visualization, Writing – original draft, Writing – review & editing. QX: Conceptualization, Methodology, Supervision, Writing – review & editing.
